# Precision medicine in cardiorenal and metabolic diseases with routinely collected clinical data: a novel insight

**DOI:** 10.1093/pcmedi/pbac025

**Published:** 2022-09-24

**Authors:** Sheyu Li, Qingyang Shi, Valentyn Litvin, Charles F Manski

**Affiliations:** Department of Endocrinology and Metabolism, MAGIC China Centre, Cochrane China Centre, Chinese Evidence-based Medicine Centre, West China Hospital, Sichuan University, Chengdu 610041, China; Department of Endocrinology and Metabolism, MAGIC China Centre, Cochrane China Centre, Chinese Evidence-based Medicine Centre, West China Hospital, Sichuan University, Chengdu 610041, China; Department of Economics, Northwestern University, Evanston, IL 60208, USA; Department of Economics and Institute for Policy Research, Northwestern University, Evanston, IL 60208, USA

Dear Editor,

Evidence-based medicine guides modern clinical practice. Quantitative evidence synthesis facilitates clinical decision-making after comprehensive interviews between clinicians and patients. Individualised concern is always critical, not only for the diversity of patients’ values and preferences, but also for questioning whether the treatment response of the individual is in line with the population. Precision medicine aims to choose the right treatment for each person.

The success of precision medicine in oncology relies on the biological progression that a particular genetic mutation determines the development and treatment response of the neoplasm. However, patients with cardiorenal and metabolic diseases have thus far benefited much less from genetic or omics technology. Although genetics and metabolic parameters show an association with individualised drug response, only a few clinicians have changed their practice.^1^ This suggests that genetic factors are associated with but may not dominantly determine drug response in patients with cardiorenal and metabolic diseases.^2^ Turning back to routinely collected clinical data, which has supported decision-making by clinicians for more than a thousand years, could be helpful.

Well-conducted randomised controlled trials provide high-certainty evidence for clinicians and patients during their daily practice.^3^ Subgroup analyses, now routine in reporting results, help identify potential treatment response diversity in patients with different covariates. However, most of them fail to reach statistical significance or clinical credibility given a large number of covariates of interest but a limited number of trial participants. Without credible support from subgroup analyses, clinicians have to treat people as if all respond equally to a drug with the same relative effect. Even when some independent subgroup analyses yield fruitful results, clinicians can find it difficult to interpret this information quantitatively for a single patient with a particular combination of these covariates.

Systematic review and meta-analysis summarise the evidence from randomised trials to support clinical practice guidelines. Without individual patient data in most cases, however, meta-analysis relies on summary effects of the published trials that represent the marginal effects on the trial population and corresponding distribution of patients’ characteristics. The pooled effect of a meta-analysis barely explains the heterogeneity of treatment effects among different groups of people when the study-level subgroup analyses or meta-regressions are subject to ecological bias (*i.e*. aggregation bias).

Data sharing of completed randomised controlled trials allows meta-analyses at the individual patient level to categorise people based on their routinely measured parameters. Statisticians and epidemiologists developed a buddle fashion approach to achieve this. Vo and colleagues addressed case-mix heterogeneity of treatment response by direct standardisation or inverse probability weighting to a target population.^4^ Coincidentally, Dahabreh and colleagues proposed a framework to transport inferences from the trial participants to a target population using either outcome regression or inverse probability weighting requesting five assumptions.^5^ Both methods require individual patient data in all included trials. When individual patient data is available in some but not all trials, Phillippo and colleagues’ multilevel meta-regression linked statistical parameters for trials with aggerate data to the parameters for trials with individual data by integrating the covariates’ distribution to form the likelihood, assuming the consistency of parameter distributions across trial populations.^6^ This assumption is reasonable in some cases but strong. The number and proportion of the available individual patient data heavily affect the degree of validity of estimations and restricted the use of this approach.

These novel approaches work, but they require sharing of at least some individual patient data of the randomised controlled trials and also strong statistical assumptions. In 2017, the International Committee of Medical Journal Editors (ICMJE) released a statement regarding data sharing policy, and some medical journals requested that authors upload individual-level patient data to accompany their articles.^7^ The Blood Pressure Lowering Treatment Trialists’ Collaboration (BPLTTC) group set a good example by integrating the individual patient data of most large trials for hypertension.^8^ Nevertheless, this data sharing was restricted within the collaborating team and the trial data remain mainly inaccessible to the public or research peers outside the group. Publishers and researchers have to await respones to their increasing requests for data-sharing until the solutions to various difficult concerns, including but not restricting to patient privacy, technical barriers, and conflicts of interest.

Strong assumptions facilitate statistical inference, but imply flawed conclusions if researchers ignore their violation. These violations happen but are often ignored in clinical practice. For example, traditional meta-analysis deals with heterogeneity between studies by assuming a random-effect distribution of treatment effects, typically following a normal distribution. However, several sources of heterogeneity exist and are not necessarily normally distributed. When this is the case, the pooled overall effect does not have a clear clinical interpretation from a patient-centred perspective.^9^

Our recent working paper applies partial identification, a credible method that has served econometrics for decades, to precision medicine utilising routinely reported subgroup analyses of binary data in randomised controlled trials.^10^ Partial identification analysis makes relatively weak, reasonable assumptions and uses them to generate bounds (*i.e*. interval or set value) on the effects of interest.^11^^,^^12^ The basic idea is to start from set-valued identification regions obtained in the absence of assumptions and then impose realistic assumptions to shrink the regions to be sufficiently informative or yield clinically useful conclusions.

Applying the method to a large randomised controlled trial of empagliflozin in adults with type 2 diabetes at high cardiovascular risk, the team selected three patient covariates (age, sex, and glycated haemoglobin [HbA1c] at baseline) that may impact response to the drug empagliflozin. Each covariate allows the anticipation of the conditional treatment effect, and we call these short outcomes. For a particular patient with a certain combination of several binary covariates, combining the corresponding short outcomes implies long outcomes, which is the individualised treatment effect. Without any assumptions, the long outcome in the given example provided null information regarding the rate of major cardiovascular events (MACE, the primary outcome of the trial) in individuals with different combinations of the three covariates. Imposing bounded-variation assumptions can make the results meaningful to clinicians. Bounded-variation assumptions restrict the potential magnitude of the effects of interest and the degree to which the effects can vary with different patients’ covariates. We propose five types of bounded-variation assumptions that could be helpful for medical estimates (Table 1). In the preliminary exercise, we assumed that a single covariate could only limitedly contribute to the drug response and the variation could be no larger than 0.05. The analyses showed that older males with lower HbA1c and older females with higher HbA1c without receiving empagliflozin are at the highest risk of MACE, while young males with higher HbA1c and young females with lower HbA1c may be at the lowest risk without taking the drug.

**Table 1:** Examples of bounded-variation assumptions.

Bounded-variation assumption	Description
Bound on the rate/risk of treatment outcome	The range between the minimal and maximal rates/risks of the outcome, reflecting the possible border of natural incidence.
Bound on absolute difference across covariates	The range between the minimal and maximal absolute rate/risk differences of outcomes in people with different covariates, reflecting the largest possible absolute magnitude of the covariate impact on the outcome.
Bound on relative difference across covariates	The range between the minimal and maximal rate/risk ratios of outcome in people with different covariates, reflecting the largest possible relative magnitude of the covariate impact on the outcome.
Bound on absolute difference across interventions	The range between the minimal and maximal absolute rate/risk differences of outcome in people receiving different interventions, reflecting the largest possible absolute magnitude of the intervention impact on the outcome.
Bound on relative difference across interventions	The range between the minimal and maximal rate/risk ratios of outcome in people receiving different interventions, reflecting the largest possible relative magnitude of the intervention impact on the outcome.

This approach does not require individual patient data, but it asks clinicians to understand bounded-variation assumptions and impose them based on clinical knowledge. The bounded variation represents the largest possible range of rate or effect size across treatment or covariates, possibly ranging from 0 to ∞. When assuming bounded variation across treatments, the clinicians should consider the theoretically maximal influence of a single patient character or an intervention on the effect size. Our illustrative exercise assumed a narrow-bounded variation to test the methodological feasibility of the approach. In practice, pooling analysis of multiple trials using a wider bounded variation could also yield a tight bound. An improper assumption of bounded variation may lead to a null bound that is not useful for practice, especially when the heterogeneity of a population or intervention is large. To solve real-world clinical problems, our new approach is a work in progress, but it has a bright potential.

In summary, precision medicine for cardiorenal and metabolic diseases calls for the utilisation of routinely collected data in practice. Individual-level patient data from large randomised controlled trials are essential to estimate personalised treatment effects precisely, but such data are largely inaccessible. When individual patient data are unavailable, a very recent approach adopting partial identification may informatively bound personalised treatment effects using weak assumptions based on clinical knowledge.
